# Supplementary material for: Barriers to and Facilitators of the Implementation of a Micronutrient Powder Program for Children: A Systematic Review Based on the Consolidated Framework for Implementation Research
Source: Nutrients. 2023 Dec 12;15(24):5073. doi: 10.3390/nu15245073 (PMC10745920; doi:10.3390/nu15245073)
Supplement: Supplementary file 1 [file nutrients-15-05073-s001.zip › Supplementary File S1 Search strategy.pdf]

Supplementary File S1. Search strategy

Search strategy for database

**Scopus**	(TITLE ((micronutrient powder OR MNP OR micronutrient supplement OR micronutrient sprinkles OR nutrient powder OR nutrient supplement)) AND TITLE ((factors OR implementation OR challenge OR facilitators OR experience OR adherence OR acceptability OR lessons OR effect))	1932
**Pubmed**	(((micronutrient powder[Title] OR MNP[Title] OR micronutrient supplement[Title] OR micronutrient sprinkles[Title] OR nutrient supplement[Title] OR nutrient sprinkles[Title])) AND ((factors[Title] OR implementation[Title] OR challenge[Title] OR facilitators[Title] OR experience[Title] OR adherence[Title] OR acceptability[Title] OR lessons[Title] OR effect[Title])))	2212
**Web of science**	Results for (micronutrient powder OR MNP OR micronutrient supplement OR micronutrient sprinkles OR nutrient powder OR nutrient supplement) (Title) AND (factors OR implementation OR challenge OR facilitators OR experience OR adherence OR acceptability OR lessons OR effect) (Title)	2431
**Medline**	Results for (micronutrient powder OR MNP OR micronutrient supplement OR micronutrient sprinkles OR nutrient powder OR nutrient supplement) (Title) AND (factors OR implementation OR challenge OR facilitators OR experience OR adherence OR acceptability OR lessons OR effect) (Title)	1721
**Embase**	(micronutrient powder:ti OR MNP:ti OR micronutrient supplement:ti OR micronutrient sprinkles:ti OR nutrient supplement:ti OR nutrient sprinkles:ti) AND (factors:ti OR implementation:ti OR challenge:ti OR facilitators:ti OR experience:ti OR adherence:ti OR acceptability:ti OR lessons:ti OR effect:ti)	815
